# Strategies for the drug development of cancer therapeutics

**DOI:** 10.3389/fphar.2025.1656012

**Published:** 2025-09-30

**Authors:** Hongyan Liu, Yanpin Ma, Wenjuan Chen, Xinyu Gu, Jiachun Sun, Penghui Li

**Affiliations:** ^1^ Henan Key Laboratory of Cancer Epigenetics, Cancer Institute, The First Affiliated Hospital, College of Clinical Medicine, Medical College of Henan University of Science and Technology, Luoyang, China; ^2^ Department of Infectious Diseases, The First Affiliated Hospital, College of Clinical Medicine, Henan University of Science and Technology, Luoyang, Henan, China; ^3^ Department of Gastrointestinal surgery, The First Affiliated Hospital, College of Clinical Medicine, Henan University of Science and Technology, Luoyang, Henan, China

**Keywords:** cancer, drug development, omics strategies, bioinformatics, networkpharmacology, molecular dynamics simulation

## Abstract

Cancer is a global health threat, with its treatment modalities transitioning from single therapies to integrated treatments. This paper systematically explores the key technological systems in modern cancer treatment and their application value. Modern cancer treatment relies on four core technological pillars: omics, bioinformatics, network pharmacology (NP), and molecular dynamics (MD) simulation. Omics technologies integrate various biological molecular information, such as genomics, proteomics and metabolomics, providing foundational data support for drug research. But the differences in data and the challenges of integrating it often lead to biased predictions, and that’s a big limitation for this technology. Bioinformatics utilizes computer science and statistical methods to process and analyze biological data, aiding in the identification of drug targets and the elucidation of mechanisms of action. It is important to note that the prediction accuracy largely depends on the algorithm chosen. Consequently, this dependence may affect the reliability of the research results. NP, based on systems biology, studies drug-target-disease networks, revealing the potential for multitargeted therapies. That said, this method may overlook important aspects of biological complexity, such as variations in protein expression. This oversight can lead to overestimating the effectiveness of multi-targeted therapies, resulting in false positives in efficacy assessments, which somewhat limits its practical usefulness. MD simulation examines how drugs interact with target proteins by tracking atomic movements, thus enhancing the precision of drug design and optimization. Nevertheless, this technology faces practical challenges, such as high computational costs and sensitivity of model accuracy to the parameters of the force field. The synergistic application of these technologies significantly shortens the drug development cycle and promotes precision and personalization in cancer therapy, bringing new hope to patients for successful treatment. However, researchers still face challenges like the variability of data. Future efforts need to use Artificial Intelligence (AI) to establish standardized data integration platforms, develop multimodal analysis algorithms, and strengthen preclinical-clinical translational research to drive breakthrough advancements in cancer treatment. With the ongoing technological improvements, the vision of personalized medicine—tailored treatments based on individual patient characteristics—will gradually be realized, significantly enhancing treatment efficacy and improving patients’ quality of life.

## 1 Introduction

Cancer is a major global health threat, and the development of effective treatment methods is a core issue in the medical field (Zaimy et al., 2017). Currently, cancer treatment faces three main challenges: limitations of traditional drug development models, inherent flaws of single-target drugs, and the complexity of tumor mechanisms. Regarding traditional drug development, existing models struggle to address tumor complexity. Specifically, single-target drugs are often limited by insufficient efficacy, rapid development of resistance, and significant side effects (Han Li and Chen, 2015; Colonna, 2025). These limitations underscore the urgency of developing new therapeutic strategies. The drug discovery and development (DDD) process encounters significant challenges. It is complex and resource-intensive, requiring meticulous monitoring and detailed data analysis at each stage. This leads to lengthy development cycles and high costs (Wu et al., 2025). To overcome these difficulties, multidisciplinary strategies have recently shown significant promise. By integrating the latest technologies such as omics, bioinformatics, NP, and MD simulation, this approach offers a novel method for cancer drug development and promises to drive breakthroughs in anti-cancer treatment.

**FIGURE 1:**
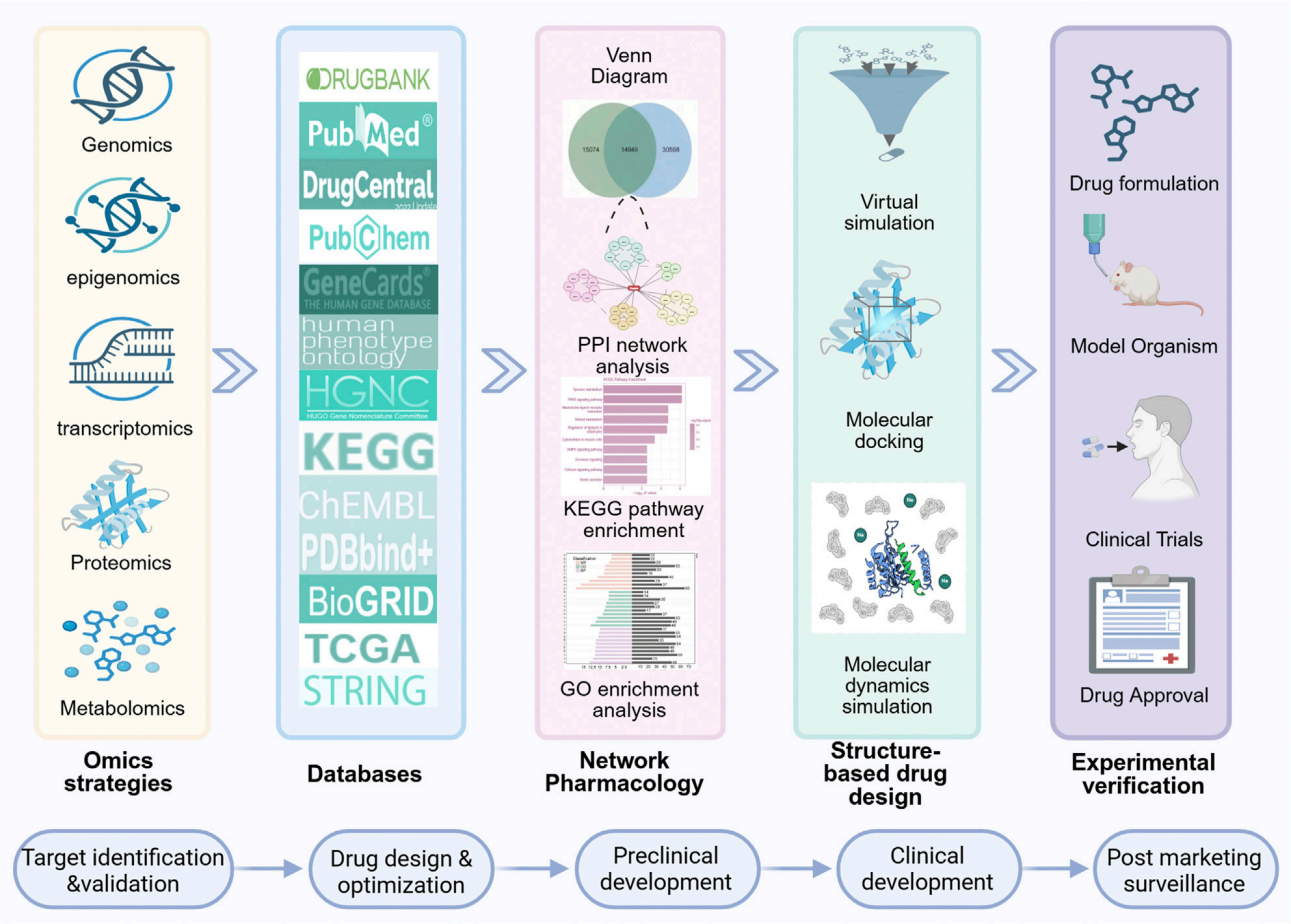
A concise overview of the drug development process utilizing cutting-edge technology. Initially, omics and database data are integrated and analyzed to construct a PPI network using NP approaches. Subsequently, KEGG pathway and GO enrichment analyses are performed. Drug design and screening are then executed via molecular docking, VS techniques and MD simulation. Finally, experimental validation is conducted, followed by the submission of new drug applications to relevant regulatory authorities. The general steps in drug development include target identification and validation, drug design and optimization, preclinical development, clinical development, and post marketing surveillance.

Omics technologies (such as genomics and proteomics) reveal disease-related molecular characteristics through high-throughput data, but using them is challenging due to data heterogeneity and a lack of standardization (Liu X. et al., 2024). For example, while compound screening based on the Chemical Entities of Biological Interest (ChEBI) database identified targets like TREM1 and MAPK1, incomplete data made it harder to validate the results (Alibakhshi et al., 2023). Bioinformatics utilizes omics data via algorithms, aiding target identification and elucidation of mechanisms (Mallick et al., 2017; Gudivada and Amajala, 2025). For instance, a Clustered Regularly Interspaced Short Palindromic Repeats–CRISPR-associated protein 9 (CRISPR–Cas9) functional genomics screen of 324 cancer cell lines prioritized targets by integrating genomic biomarkers, including microsatellite instability (MSI). However, algorithms struggle to fully grasp the complexity of biological systems, which can lead to prediction errors (Behan et al., 2019). Building on these insights, NP constructs drug-target-disease networks through systems biology methods, facilitating the development of multi-target therapeutic strategies. Research indicates that multi-target xanthine oxidase inhibitors can synergistically lower uric acid production and reduce adverse reactions (Yi et al., 2023). Meanwhile, natural multi-target neuraminidase inhibitors exert antiviral effects by regulating pathways such as Toll-like receptor 4 (TLR4) and Interleukin-6 (IL-6), significantly broadening the understanding of drug action mechanisms (Cao et al., 2024). However, their predictive performance heavily depends on experimental validation. For example, verifying how parthenolide (PTL) affects breast cancer (BC) pathways requires molecular docking, MD simulation, and both *in vivo* and *in vitro* experiments; without such validation, false-positive results may occur (Shi et al., 2024). The final stage involves molecular docking and MD simulation to improve the accuracy of drug design through atomic-level interaction analysis. For instance, Molecular Mechanics/Poisson–Boltzmann Surface Area (MM/PBSA) calculations show that phytochemicals have a binding free energy of −18.359 kcal/mol with Asialoglycoprotein receptor 1 (ASGR1), indicating strong binding affinity (Gao et al., 2023). Additionally, optimization methods for tankyrase inhibitors can guide structural improvements of new anti-cancer drugs (Kamal et al., 2014). However, simulations can be sensitive to force field settings and are difficult to replicate under real-life conditions, limiting their clinical translation potential (Chen et al., 2024). Therefore, more *in vivo* and *in vitro* experiments are still needed (Figure 1). The research by the Bao team is a typical case. They used NP to screen the action targets of Formononetin (FM). Then, they calculated the network contribution index through mathematical formulas to determine the core components. They also analyzed differentially expressed genes in liver cancer using the Cancer Genome Atlas (TCGA) database. Subsequently, they evaluated how well FM binds to its targets using molecular docking. They confirmed the stability of FM binding to glutathione peroxidase 4 (GPX4) through metabolomics analysis and MD simulation using ultra-performance liquid chromatography–tandem mass spectrometry (UPLC-MS/MS). Finally, laboratory and animal tests showed that FM causes DNA damage, which stops the cell cycle and regulates glutathione metabolism to inhibit the p53/xCT/GPX4 pathway, thereby inducing ferroptosis and suppressing liver cancer progression (Bao et al., 2025).

The development of cancer therapeutic drugs is entering a new era of precision and personalization. By integrating omics strategies, bioinformatics analysis, NP, and MD simulation, researchers can systematically reveal the molecular mechanisms of cancer occurrence and development. This multidisciplinary research approach has three main advantages: firstly, it can efficiently identify potential novel therapeutic targets (Choubey and Jeyaraman, 2016; Khan and Khan, 2025; Yanzhang et al., 2025); secondly, it can optimize and improve existing drugs at the molecular level (Duan et al., 2023); finally, it provides a scientific basis for developing personalized treatment plans (Matchett et al., 2017). These breakthroughs not only bring new treatment hope to cancer patients but also open innovative pathways for future medical research. As research continues to deepen, the vision of personalized medicine is gradually being transformed into reality, promising to significantly enhance treatment efficacy and improve the quality of life of cancer patients. The future outlook focuses on multimodal data integration, AI-driven high-throughput screening (HTS), and the development of standardized platforms. By developing algorithm-based methods to optimize multi-targeted drug design and enhancing translational research from preclinical to clinical stages, we will aim to address challenges—such as genomic, proteomic, and clinical data diversity and off-target effects (Alibakhshi et al., 2023). Only through interdisciplinary collaboration and technological innovation can we overcome current hurdles and advance cancer treatment to be more precise and personalized.

## 2 Technologies

### 2.1 Omics strategies

Omics data, the product of high-throughput technologies, originate from various fields such as genomics, proteomics and metabolomics, and holds significant value in drug research (Zhang et al., 2024). Specifically, genomics helps to identify disease-related genes by analyzing massive data, promoting targeted drug development and personalized medicine (Schmidt et al., 2022). Meanwhile, proteomics elucidates the role of proteins in diseases by analyzing protein structures, providing a basis for drug design (Lee et al., 2011; Gomase et al., 2025). Metabolomics studies small molecule metabolites, offering key clues for discovering cancer treatment targets (Schmidt et al., 2021). There are significant differences in the predictive capabilities and application value of different omics in the field of oncology, but there’s still not enough evidence to directly compare the predictive abilities of different cancer types. Right now, research is focusing on the need for multi-omics integration to help speed up drug development by working with other fields (He et al., 2023; Pan et al., 2025).

Genomics technology, as one of the core pillars of modern biomedical research, plays an increasingly prominent role in the development of cancer drug targets. It systematically studies the complete genetic information of organisms to reveal the structures, functions and mechanisms of genes in disease occurrence and development (Martínez-Jiménez et al., 2020). With the rapid development of high-throughput sequencing technology, genomics has gradually transitioned from basic research to clinical applications, providing strong technical support for the precise diagnosis and targeted treatment of cancer (Mukherjee, 2019). Among its core technologies, mainly including DNA microarrays and next-generation sequencing (NGS) methods (Dong et al., 2015), the former is a high-throughput technology based on the principle of nucleic acid complementary pairing, where known DNA probes are fixed on a support to form an array (Dang et al., 2014). The sample to be tested is labeled and hybridized with the probes, determining the presence and expression levels of DNA/RNA in the sample through signal detection (Zhu et al., 2006). This technology can be used for gene expression profiling, genotyping and disease research. It is characterized by high throughput, sensitivity and specificity, whereas it requires high sample quality and data analysis and is relatively costly (Liu Y. et al., 2024). NGS technology integrates whole genome sequencing (WGS), whole exome sequencing (WES) (Enko et al., 2023)and genome-wide association studies (GWAS) (Jiang et al., 2014). In WGS can cover the entire genome, providing the most comprehensive information on genetic variations, such as single nucleotide variations (SNVs), insertions/deletions (INDELs), copy number variations (CNVs), and structural variations (SVs) (Li et al., 2021). In cancer research, WGS helps discover driver mutations, tumor heterogeneity and clonal evolution patterns, providing key clues for targeted drug development (Egan et al., 2012). In contrast, WES focuses on the exonic regions that encode proteins (Georget and Pisan, 2023). Although its coverage is smaller, it is more cost-effective and the data volume is more controllable, thus it is widely applied in clinical research. Through WES, researchers can efficiently identify functionally relevant mutations associated with cancer, such as variations in key genes like tumor protein 53 (TP53), epidermal growth factor receptor (EGFR) and KRAS, which are often important targets for targeted drug development (Witkiewicz et al., 2015). GWAS analyzes a large number of single nucleotide polymorphisms (SNPs) in the genome to determine whether these SNPs are associated with specific traits or diseases, thus locating genes or genomic regions that may be related to diseases (Chang et al., 2018). In addition, the continuous development of genomics technologies, such as CRISPR gene editing (Cruz and Freedman, 2018), induced pluripotent stem cells, and organoids, is constantly expanding the range of available tools for drug discovery (Boreström et al., 2018). It is important to note that while these genetic technologies can identify how genetic variations relate to drug responses, drug responses are influenced by a bunch of other factors, such as protein expression, how the body metabolizes drugs, and epigenetic regulation. Just relying on genomic data might not really show how complex drug responses can be (Doble et al., 2013).

Proteomics, as an important research field in the post-genomic era, plays an irreplaceable role in elucidating disease mechanisms and drug development (Li et al., 2014). Compared to genomics, it can more directly reflect the dynamic changes in cellular life activities. However, the high cost of protein testing technology and its broad dynamic range present unique challenges. Additionally, accurately quantifying low-abundance proteins is particularly difficult (Fall et al., 2025). Proteomics research mainly includes three key stages: sample preparation, technology application and data analysis (Bose et al., 2019). This field aims to achieve systematic identification and quantification of the entire proteome, elucidating the biological functions of proteins, providing a broad application value in biological target discovery and drug development (He and Chiu, 2003; Savino et al., 2012). In terms of technology, proteomics research faces a dual choice between traditional methods and emerging technologies. Traditional protein detection methods, such as enzyme-linked immunosorbent assay (ELISA) (Aydin, 2015), chemiluminescence immunoassay (CLIA) (Hayrapetyan et al., 2023), and immunohistochemistry (IHC) (Ramos-Vara, 2005), have significant limitations when processing complex proteomes. Modern high-throughput proteomics technologies mainly include MS-based techniques, antibody/antigen arrays, and aptamer-based detection (Liu Y. et al., 2024). MS-based techniques involve sample preprocessing and the liquid chromatography analysis of proteins and their modifications, making them the preferred method for biomarker discovery (Wei and Li, 2009). Common subtypes include liquid chromatography-mass spectrometry (LC-MS), matrix-assisted laser desorption/ionization time-of-flight mass spectrometry (MALDI-TOF MS) (Yang and Chien, 2000), matrix-assisted laser desorption/ionization immunohistochemistry mass spectrometry (MALDI-IH-MS) (Taylor et al., 2025), and evaporative light scattering detection-assisted liquid chromatography-mass spectrometry (ELSD-LC-MS) (Cao et al., 2018). Antibody/antigen array methods utilize immobilized antibodies to detect proteins, which are widely applied but have a limited dynamic range. Aptamer-based detection has high affinity and specificity but is limited by the difficulty of developing high-quality aptamers (Liu Y. et al., 2024). These technologies demonstrate significant application value in cancer mechanism research, immunotherapy evaluation, target discovery, and new drug development (Sanchez-Carbayo, 2006; Sanchez-Carbayo et al., 2006; Uemura and Kondo, 2015; Kwon et al., 2021).

Metabolomics, as an important component of systems biology, was first proposed by Steven Oliver in 1998 and has since become a key method for studying the interactions between genes and the environment (Rochfort, 2005). This discipline focuses on high-throughput studies of all small molecules (50–1,500 Da) in cells, biological fluids, tissues, or organisms, collectively referred to as metabolites, which have diverse physicochemical characteristics and dynamic abundance ranges (Johnson et al., 2016). Metabolites can track real-time changes in metabolic products, which makes it easier to see health and disease states. They have distinct benefits in assessing drug metabolism kinetics. They also help in evaluating toxic responses. However, metabolomics data can be easily affected by factors such as the environment, diet, and individual physiological states, so just be careful when interpreting the data (Doble et al., 2013; Martinez-Lozano Sinues et al., 2014). With the development of omics technologies, metabolomics plays an increasingly important role in the pharmaceutical industry and clinical applications, especially in drug development and treatment. Metabolomics research mainly relies on two major technological platforms: data acquisition platforms and data analysis platforms (Chen et al., 2013). The core technologies of data acquisition platforms include nuclear magnetic resonance (NMR) (Gowda and Djukovic, 2014) and MS (Liu and Wen, 2024) related technologies. NMR technology (such as LC-NMR and gas chromatography (GC)-NMR) can perform the structural identification and quantitative analysis of metabolites, providing detailed molecular structure information (Gebregiworgis and Powers, 2012; Aminov, 2022). While MS-related technologies are characterized by high sensitivity and throughput that allow the simultaneous analysis of various metabolite components (Liu Y. et al., 2024). These include LC-MS (Chen et al., 2023), GC-MS (Ren et al., 2013; Beale et al., 2018), and capillary electrophosis (CE)-MS (Seyfinejad and Jouyban, 2022), etc. Currently, metabolomics mainly employs four basic techniques: LC-MS, GC-MS, CE-MS and NMR. Statistics show that LC-MS has the highest usage rate (83%), followed by GC-MS (30%) and NMR (26%) (Weber et al., 2017). These technologies show great potential in phenotypic classification, physiological state monitoring, disease diagnosis, treatment response evaluation, as well as biomarker discovery (Naz et al., 2013). However, each technology has its advantages and limitations: LC-MS is suitable for analyzing most metabolites; GC-MS excels in detecting volatile compounds; NMR can provide structural information without destroying samples; and CE-MS is suitable for the separation and analysis of charged metabolites (Liu Y. et al., 2024). Therefore, in practical research, the selection of technology should be based on specific needs, considering factors such as the physicochemical properties and concentration ranges of target metabolites.

In addition, advanced spatial transcriptomics platforms (such as Visium and Xenium) allow for high-throughput detection of gene expression while retaining spatial information. These platforms overcome the limitations of traditional single-cell sequencing methods, which often lose spatial structure (Quan et al., 2023; Liu Q. et al., 2024). By using bioinformatics tools like Seurat, researchers can normalize data, perform dimensionality reduction, identify spatial patterns of gene expression, and integrate with single-cell data. This approach helps systematically analyze complex biological processes, such as the tumor microenvironment, thereby offering new insights for cancer drug development (Satija et al., 2015).

### 2.2 Bioinformatics analysis

Bioinformatics is an interdisciplinary field that integrates methods and technologies from computer science, mathematics and statistics to acquire, store, manage, analyze, and interpret biological data from genomics, transcriptomics, proteomics, etc., thereby revealing the complexity and functional mechanisms of biological systems and addressing related issues in biology and medicine (Merrick et al., 2011; Simonian, 2021; Shahjahan et al., 2024). The rapid development of this discipline began with the proposal of the Human Genome Project by American scientist Robert Sinsheimer and its subsequent completion, marking the increasing importance of bioinformatics in life sciences (Templeton, 1998). In cancer treatment, bioinformatics helps researchers to accurately identify potential drug targets and prognostic biomarkers by integrating multi-omics data, deeply analyzing and validating the mechanisms of action of drugs and predicting drug resistance, which can significantly improve the precision and effectiveness of treatments (Dong et al., 2019; Rehman et al., 2020; Fang et al., 2022). The bioinformatics tools and technologies in drug discovery mainly include biological databases (Table 1), computer-aided drug design (CADD) and omics technologies (see the omics strategies section) (Zhang et al., 2025).

**TABLE 1:** Commonly used repositories related to human diseases, drug targets, omics data, and biological networks.

Types	Database name	Description	Web link	Ref.
Diseases	OMIM	An exhaustive, credible, and current compendium of human genes and genetic disorders	http://www.omim.org/	Hamosh et al. (2021)
	PALGA	A repository containing histopathological and cytopathological data was established	https://www.palga.nl	Becherer et al. (2019)
	DisGeNET	A comprehensive database integrating information on human genetic associations with disease	https://disgenet.com/	Hu et al. (2025)
	CIViC	A community-driven database for clinical interpretation of cancer mutations that facilitates precision cancer medicine	https://civicdb.org/	Krysiak et al. (2023)
Drug Targets	DrugBank	A comprehensive web-based database of molecular information about drugs, drug mechanisms, drug interactions, and drug targets	https://www.drugbank.ca/	Knox et al. (2024)
	TTD	A database offering details on known therapeutic protein and nucleic acid targets, as well as associated targeted diseases	http://db.idrblab.net/ttd/	Zhou et al. (2024b)
	PubChem	An open database containing chemical structures and their bioassay results	http://pubchem.ncbi.nlm.nih.gov	Wang et al. (2017)
	ChEMBL	An open database containing functional and ADMET information on bioactive compounds of many classes of drugs	https://www.ebi.ac.uk/chembldb	Zdrazil et al. (2024)
Drug Targets	GDSC	A large public database focused on providing data on the sensitivity of cancer cells to drugs as well as information on molecular markers	https://www.cancerrxgene.org/	Yang et al. (2013)
	PharmGKB	A comprehensive pharmacogenomics database focused on collecting and collating the effects of genetic variants on drug response	https://www.pharmgkb.org/	Barbarino et al. (2018)
	LINCS	A database for studying disease mechanisms and driving drug discovery	https://lincsproject.org/	Evangelista et al. (2022)
	BindingDB	A publicly available database focused on collecting data on the interaction of drug target proteins with small molecules and their affinity	https://www.bindingdb.org/	Evangelista et al. (2022)
	CellPhoneDB	A database specifically designed to infer communication between cells by analyzing ligand-receptor interactions using transcriptomic data from single-cell RNA sequencing or spatial transcriptomic sources	https://www.cellphonedb.org/	Efremova et al. (2020)
	IUPHAR/BPS Guid to PHARMACOLOGY	An open database providing pharmacological, chemical, genetic, functional, and pathophysiological data on approved and experimental drug targets	https://www.guidetopharmacology.org/	Harding et al. (2024)
	PDSP Ki Database	A public database that provides data on the affinity (Ki) of drugs to a variety of molecular targets	https://pdspdb.unc.edu/kidb2/kidb/web/	Baker et al. (2015)
	DrugCentral	An open drug resource that provides drug structure, biological activity, regulatory information, pharmacological actions, and indications	https://drugcentral.org/	Ursu et al. (2019)
Omics Data	GEO	A public database for storing and sharing high-throughput genomic data	https://www.ncbi.nlm.nih.gov/geo/	Barrett et al. (2013)
	TCGA	A database containing clinical data, genomic mutations, mRNA expression, miRNA expression, methylation, and more for a variety of human tumors	https://cancergenome.nih.gov/	Colaprico et al. (2016)
	CCLE	A compilation database of gene expression, chromosome copy number, and massively parallel sequencing data from 947 human cancer cell lines	https://sites.broadinstitute.org/ccle	Dutil et al. (2019)
	ENCODE	A database containing regions such as transcription, transcription factor association, chromatin structure, and histone modifications	https://www.encodeproject.org/	Jou et al. (2019)
	GWAS Catalog	A publicly available database that includes the results of genomic association studies (GWAS) worldwide	https://www.ebi.ac.uk/gwas/	Sollis et al. (2023)
	COSMIC	A database that manages comprehensive information on somatic mutations in human cancers	http://www.sangerac.uk/cosmic	Dutil et al. (2019)
	ICGC	A detailed genomic database of more than 50 cancers	https://dcc.icgc.org	The ICGC/TCGA Pan-Cancer Analysis of Whole Genomes Consortium (2020)
Omics Data	cBioPortal	An open database that supports the browsing and analysis of multidimensional cancer genomic data	https://www.cbioportal.org	Cerami et al. (2012)
	UCSC Xena	A database that integrates multiple large cancer research projects and supports visualization, analysis, and downloading of data	https://xena.ucsc.edu	Wang et al. (2022b)
	GEPIA2	A web server for large-scale expression analysis and interaction analysis	http://gepia2.cancer-pku.cn	Tang et al. (2019)
	GSCA	A gene set cancer analysis platform integrating TCGA, GTEx, GDSC, and CTRP.	https://guolab.wchscu.cn/GSCA	Liu et al. (2023a)
	GDAC Firehose	A tool for analyzing large-scale genome and proteome data from TCGA.	https://gdac.broadinstitute.org	Rau et al. (2019)
	CPTAC	A clinical proteomics tumor analysis database	https://proteomics.cancer.gov/programs/cptac	Li et al. (2023)
	CVCDAP	A web-based cancer virtual cohort data analysis platform that integrates TCGA and CPTAC data	https://omics.bicancer.org/cvcdap/home.do	Guan et al. (2020)
	UALCAN	An interactive web-based resource platform for analyzing cancer omics data	http://ualcan.path.uab.edu	Chandrashekar et al. (2022)
	TCPA	A resource for acquiring and analyzing functional proteomics in cancer	http://tcpaportal.org/tcpa	Chen et al. (2019b)
Omics Data	CancerProteome	A resource to functionally decipher the proteome landscape of cancer	http://biobigdata.hrbmu.edu.cn/CancerPro-teome	Lv et al. (2024)
	SPOKE	A multi-layer network integration, biomedical knowledge map repository	https://spoke.ucsf.edu/	Morris et al. (2023)
	Tri©DB	An open source cancer precision medicine knowledge base based on the gene-disease-treatment relationship	http://www.biomeddb.org/	Jiang et al. (2023)
	GTEx	A database for studying genetic variation and gene expression	https://www.gtexportal.org/home/	(2013)
	HMDB	A database and analysis tool that provides metabolites of humans and other species	http://www.hmdb.ca/	Wishart et al. (2009)
	METLIN-CCS	One contains more than 960,000 compounds and is currently the largest secondary mass spectrometry database	https://metlin.scripps.edu/	Baker et al. (2024)
	mzCloud	A mass spectrometry database that provides high quality multistage mass spectrograms covering a wide range of compound information	https://www.mzcloud.org/	Shi et al. (2022)
	MassBank	A database that provides mass spectra of metabolites and small molecule compounds	http://www.massbank.jp/	Horai et al. (2010)
	UniProt	A comprehensive protein database that provides information on the known sequence and function of proteins on a global scale	https://www.uniprot.org/	UniProt Consortium (2021)
Omics Data	PDB	A database that stores the 3D structures of proteins	https://www.rcsb.org/	Berman et al. (2000)
	InterPro	A database that provides information on the classification of protein families and domains	https://www.ebi.ac.uk/interpro/	Paysan-Lafosse et al. (2023)
	SMART	An online analysis tool for protein domain identification and annotation	http://smart.embl-heidelberg.de/	Schultz et al. (2000)
	Human Protein Atlas	A database that maps all human proteins using multiple omics techniques	https://www.proteinatlas.org/	Digre and Lindskog (2023)
Biological Networks	STRING	A repository of established and forecasted protein interactions	http://string-db.org/	Szklarczyk et al. (2023)
	GO	A comprehensive database of gene functions	http://www.geneontology.org/	Harris et al. (2004)
	KEGG	A database of genomes, biological pathways, diseases, drugs, and chemicals	http://www.genome,jp/kegg/	Kanehisa and Goto (2000)
	BioGRID	A repository of genetic and protein interaction biomedical interactions that archives multiple model organisms and humans	http://thebiogrid.org/	Oughtred et al. (2019)
	Reactome	A database of free biomolecular pathway knowledge	https://reactome.org	Rothfels et al. (2023)
	SIGNOR 3.0	A database containing causal relationships in signal transduction, pathway visualization	https://signor.uniroma2.it/	Lo Surdo et al. (2023)
	OmniPath	An integrated multi-source database of PTM relationships, protein complexes, and intercellular communication	https://omnipathdb.org/	Türei et al. (2016)

CADD can be divided into structure-based drug design (SBDD) and ligand-based drug design (LBDD). The core of SBDD lies in utilizing the three-dimensional structure of target proteins and the characteristics of their binding sites to promote drug discovery and design through steps such as target preparation, binding site identification, molecular docking, virtual screening (VS), and MD simulation (Vemula et al., 2023). In the absence of knowledge of the three-dimensional structure of the receptor, LBDD targets known ligands, establishing the structure-activity relationship between their physicochemical properties and activity to facilitate drug repurposing (Hirokawa, 2022). Quantitative structure-activity relationship (QSAR) and pharmacophore methods are two main techniques of LBDD (Yu and MacKerell, 2017). Additionally, due to the high cost of HTS, techniques such as molecular docking and VS provide efficient supplementary means for experimental processes, further accelerating drug development (Kontoyianni, 2017). These tools and technologies in bioinformatics lay a solid foundation for modern drug development and precision medicine.

**FIGURE 2:**
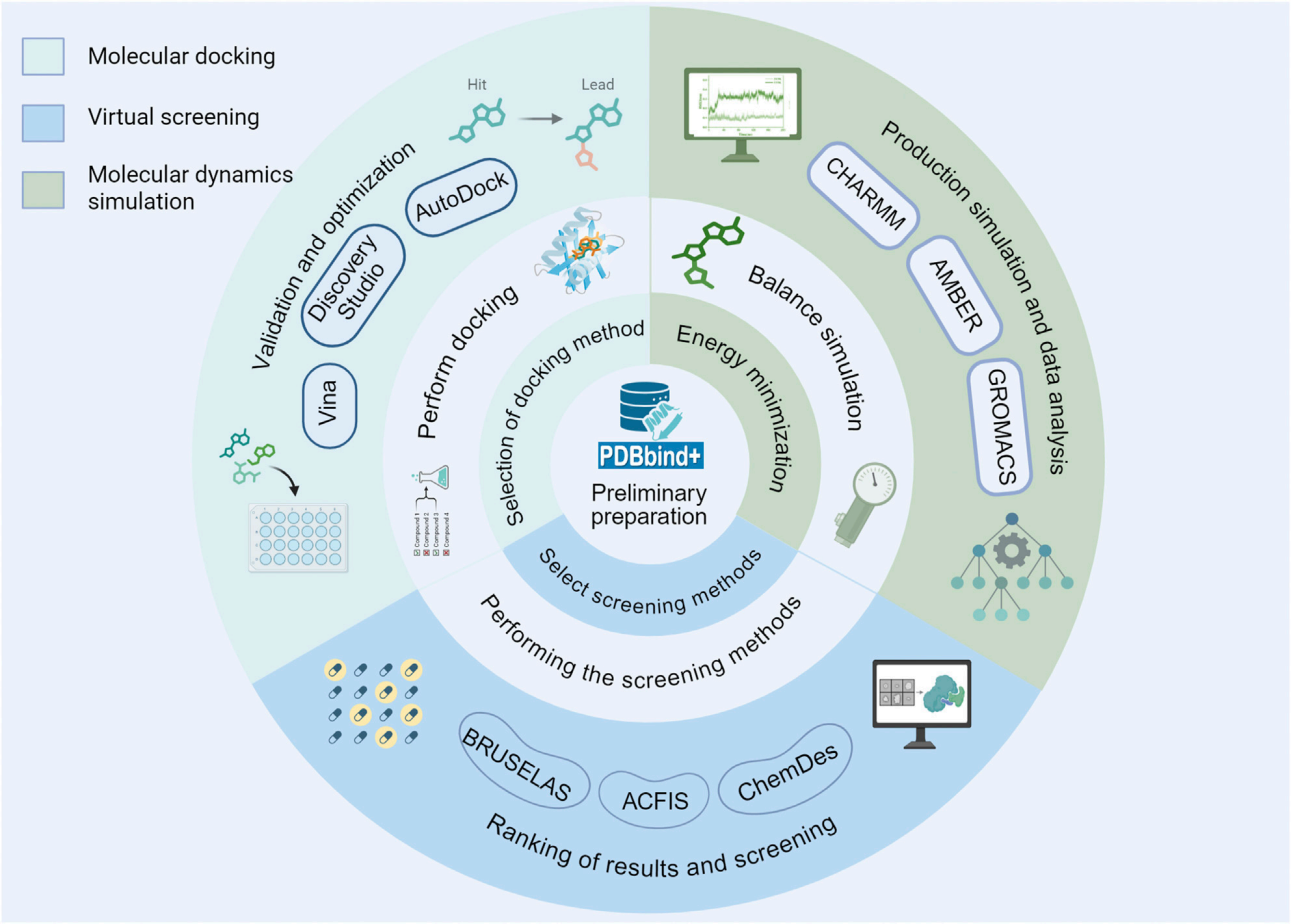
The fundamental procedures of molecular docking, VS and MD simulation involve several key stages that are essential for accurate computational modeling.

VS serves as an important complementary tool to experimental HTS, efficiently screening large compound databases and significantly enhancing the efficiency of drug discovery. Its core advantages are mainly reflected in three aspects: first, VS can efficiently process massive compound databases, screening far more compounds than traditional methods; second, computer pre-screening can significantly reduce the number of compounds that need to be validated later, significantly cutting the costs of developing complex HTS testing methods; finally, VS only requires a computer representation of the compounds, removing the need to synthesize them beforehand, giving a unique edge in exploring uncharted chemical territories (Watson et al., 2003; Banegas-Luna et al., 2018). Its process integrates target structure preparation, compound library construction, molecular docking to calculate binding energies and rank compounds, chemical structure clustering, and visual filtering to select representative compounds for experimental validation (Figure 2; Subramaniam et al., 2008; Stumpfe et al., 2012; Kontoyianni, 2017). Currently, tools such as BRUSELAS, ChemDes and ACFIS are widely used in VS (Singh et al., 2021). VS has two significant advantages over HTS: first, the number of compounds that can be screened far exceeds that of experimental methods; second, only a small number of compounds need to undergo low-throughput experimental analysis, greatly reducing the cost of developing complex HTS detection methods (Lyu et al., 2023). The unique value of VS lies in its ability to discover novel ligands, thereby avoiding off-target side effects of known ligands and expanding the functional diversity of drugs and chemical probes (Stein et al., 2020; Fink et al., 2022; Singh et al., 2023). In addition, the VS library only requires computer representation and does not need to be synthesized in advance, providing flexibility for exploring unknown chemical spaces (Manglik et al., 2016; Lyu et al., 2019; Sadybekov et al., 2020). In the drug discovery process, VS and HTS collaborate in the target selection and hit identification stages: HTS tests compound activity through experiments, while VS prioritizes potential active molecules through theoretical calculations, significantly narrowing the scale of subsequent experiments (Kontoyianni, 2017; Yan et al., 2020). Structure-based VS has become a core means of drug design, with parameters flexibly adjusted according to target characteristics, while the core protocol always revolves around docking and scoring. By predicting the interactions between ligands and protein binding pockets, VS can quickly identify candidate compounds, laying a solid foundation for subsequent optimization and clinical research (Hou and Xu, 2004; Houston and Walkinshaw, 2013; Yu et al., 2023). It is worth noting that the quality of VS results primarily depends on three key factors: the diversity of the compound library, drug-likeness, and synthetic feasibility. Although similarity-based screening methods are reliable, they may miss lead compounds with entirely new mechanisms of action. Additionally, compounds from initial screening typically need further refinement through molecular docking. They must ultimately be validated through *in vitro* experiments to rule out false positives and ensure the screening’s reliability (Li et al., 2011; Chen M. et al., 2019; Puniani et al., 2025).

Molecular docking is a prediction technology for biomolecular interactions based on the lock-and-key principle and induced fit theory, which quickly predicts the binding conformations of ligands and receptors through geometric matching and energy optimization algorithms (Patel et al., 2024). This technology originated in the 1970s, was initially used to study the interactions between proteins and small molecules, and has now expanded to complex systems such as protein-protein and nucleic acid-ligand interactions, even applicable to larger molecules like short peptides and macrocyclic compounds (Agrawal et al., 2019; Porter et al., 2019; Martin et al., 2020). Depending on the flexibility of the receptor conformation, molecular docking can be divided into rigid docking (where the receptor is fixed and only the ligand is flexible) (Amaro et al., 2018; Kamenik et al., 2021) and flexible docking (where both the receptor and ligand can undergo conformational changes), with the latter being closer to the real biological environment but having higher computational costs (Ding and Dokholyan, 2013; Park et al., 2021). Ensemble docking integrates the docking results from multiple receptor conformations, achieving a balance between efficiency and accuracy (Cavasotto, 2012). Its basic process involves three key steps: structure preparation, binding site determination, and docking analysis (Figure 2; Paggi et al., 2024). Currently, there are over a hundred docking tools, such as AutoDock (Morris et al., 2009), Vina (Trott and Olson, 2010) and Discovery studio (Jiang and Gao, 2019), which evaluate binding energies through scoring functions and select optimal binding modes using sampling programs. This technology can accurately predict the interaction forces (such as hydrogen bonds and hydrophobic interactions) between small molecule compounds and the active pockets of target proteins, accurately obtain binding conformations and binding energy data, and provide direct guidance for subsequent structural optimization. For example, previous studies have confirmed the direct binding of components such as hypaconitine in sini decoction to tumor necrosis factor-α (TNF-α) protein through molecular docking (Chen et al., 2016). Molecular docking plays an important role in the drug development process. After potential targets are initially screened from analyses such as NP, molecular docking can serve as a key virtual validation method, which is used to preliminarily assess how likely and reasonable the binding is. Molecular docking is commonly used to measure the binding affinity. Lower (more negative) values mean stronger binding (Ralte et al., 2024). Furthermore, this technology works well with MD simulation: molecular docking provides initial binding conformations, while MD reveals dynamic interaction mechanisms, and together they promote the deeper development of drug design and biomolecular research (Lei et al., 2020; Kelly et al., 2021; El Daibani et al., 2023). However, molecular docking technology still has several limitations. First, the reliability of its results greatly depends on the quality of the three-dimensional structure of the target; if there are any missing parts or flexible loops in the protein structure, it may lead to deviations in docking results. Second, compared to the initial screening of VS, detailed molecular docking (especially docking that considers protein flexibility) requires a lot of computational power and is usually not suitable for the preliminary screening of ultra-large compound libraries. In addition, the docking scoring function mainly evaluates static binding affinity, which makes it tough to accurately simulate the complex physiological environment in living organisms (such as solvation effects, entropy changes, etc.), which might result in false positives in experiments for molecules that are predicted to bind well (Gioia et al., 2017; Kochnev et al., 2024; Puniani et al., 2025).

### 2.3 Network pharmacology

NP is a research method based on the principles of systems biology, emphasizing the interactions between drugs, targets, diseases, and biological networks. Its core idea is to link drugs with molecules in biological networks (such as proteins, genes, and metabolites) to reveal the mechanisms of action of a drug and its potential side effects. In relation to traditional targeted screening, NP not only focuses on the action of single targets but also considers the interactions of multiple targets and complex pathways, thereby achieving multitargeted therapy (Hao da and Xiao, 2014; Chandran et al., 2015; Zhai et al., 2025). Conventional methods might be limited because they overlook the network’s overall regulatory effects. In the screening and development of cancer drugs, NP provides an effective strategy for integrating various data resources and computational methods. By combining machine learning (such as Bayesian inference modeling) and computational tools (such as PharmMapper and DRAR-CPI), we can efficiently process large-scale compound-target data, predict potential targets, and screen key proteins (such as mitogen-activated protein kinase 14 (MAPK14)), and identify candidate molecules with therapeutic potential. This strategy significantly reduces the guesswork in experimental screening while greatly minimizing the use of resources, offering a smart and budget-friendly solution for drug development (Guo et al., 2023; Noor et al., 2023; Puniani et al., 2025). By constructing disease-drug-pathway-target networks, researchers can identify potential drugs and their mechanisms of action. The use of omics technologies and bioinformatics further accelerates the discovery and development of new drugs (Li and Zhang, 2013).

In cancer drug screening, NP systematically analyzes the complex relationships between drugs, targets and diseases by integrating multi-source biological data (such as cancer genome maps, drug target databases, etc.). Its core workflow includes the following steps: first, collecting information on the chemical structures, pharmacological effects and target information of drugs while combining the clinical characteristics, gene expression data and pathological mechanisms of cancer to construct drug-target-disease interaction networks; second, using network analysis tools (such as Cytoscape) and algorithms to analyze the topological structure, key nodes and functional modules of the network, revealing the potential mechanisms of action of a drug and novel therapeutic targets; subsequently, through gene function enrichment analysis (such as Gene Ontology (GO) and Kyoto Encyclopedia of Genes and Genomes (KEGG) pathway analysis), clarifying the regulatory roles of target genes in biological processes, molecular functions and signaling pathways; finally, combining molecular docking techniques to validate the binding modes of drugs and targets, and further evaluating the efficacy and safety of drugs via *in vitro* cell experiments and *in vivo* animal experiments (Gogoi and Saikia, 2022; Li et al., 2022; Tian and Tang, 2022; Wang J. et al., 2022; An et al., 2024; Joshi et al., 2025). In addition, the predictive performance of NP is determined by how good the data sources are, how accurate the target analysis is, how deep the enrichment is, and later validation with molecular docking and MD simulation. All these factors work together to ensure the model’s reliability and efficiency in drug development.

In NP research, Cytoscape, as a powerful network analysis tool, provides important support for constructing and visualizing drug-target-disease networks. Its core functions are calculating topological properties such as node degree and clustering coefficient through the NetworkAnalyzer plugin, identifying closely connected gene modules using the Molecular Complex Detection (MCODE) plugin, and screening key hub genes with the CytoHubba plugin (Ma et al., 2021; Liu et al., 2025). Furthermore, network analysis algorithms play a key role in research, encompassing various types. Network construction and analysis algorithms, such as weighted gene co-expression network analysis (WGCNA), are used to identify gene network modules and key genes, while Protein-protein Interaction (PPI) network analysis algorithms construct networks based on protein interaction information, and MCODE is used to identify dense subgraphs within networks. The key node identification algorithms include betweenness centrality, degree centrality, closeness centrality, and eigenvector centrality, which help to locate the core regulatory genes or targets within networks (Zhao et al., 2022; Nazir, 2024). Network module analysis algorithms, such as Markov clustering (MCL) and Louvain clustering algorithm, are used to partition functionally related gene or protein modules (Du et al., 2014; Lim et al., 2019). Network similarity and prediction algorithms involve topological similarity analysis, node similarity-based network analysis, and prediction algorithms based on machine learning or network topology, which can be used to infer drug-target interactions or screen potential drugs (Vella et al., 2018). In recent years, the introduction of emerging algorithms like graph neural networks (GNN) has further enhanced the research capabilities of NP. For example, GNN can integrate single-cell data, learning and inferring on complex graph structures, providing more precise analytical tools for disease gene prediction and drug target identification (Li et al., 2025). The comprehensive application of these tools and algorithms not only deepens the understanding of drug mechanisms but also provides strong technical support for drug discovery and precision medicine.

### 2.4 Molecular dynamics simulation

MD simulation is a computer simulation method based on molecular force fields (such as AMBER (Wang et al., 2004), CHARMM (Vanommeslaeghe et al., 2010), OPLS, GLOMOS, and coarse-grained force fields (Wang and O'Mara, 2021)), which descrvmdibes the interaction forces between atoms (including bond lengths, bond angles, dihedrals, van der Waals forces, and electrostatic forces) and uses numerical integration algorithms (such as the Verlet algorithm) to calculate atomic motion trajectories, thereby revealing the dynamic behavior of molecular systems (Vemula et al., 2023). The application of MD simulation can be traced back to 1957 when Alder and Wainwright first used it to study simple gases (Isobe, 2008). Twenty years later, McCammon and others pioneeringly expanded MD simulation to the field of proteins (Sinha et al., 2022). Today, with the rapid development of computational technology, MD simulation can not only study single proteins but also analyze complex protein-protein/DNA/RNA complexes (Norberg and Nilsson, 2003; Martin and Frezza, 2022). In particular, breakthroughs in supercomputers (such as the Anton computer designed for MD simulation) and graphics processing units (GPUs) have significantly enhanced the scale and efficiency of simulations, making MD simulation increasingly important in biophysics, drug design, and materials science (Schmid et al., 2010; Vermaas et al., 2021).

The key steps of MD simulation include system construction, energy minimization, equilibrium simulation, and production simulation. First, in the system construction phase, the initial structure of the target molecule (such as a protein-ligand complex) must be determined, and environmental factors such as solvent molecules (e.g., water) and ions must be added. This step typically relies on existing structural information from protein databases (such as Protein Data Bank (PDB)). If the 3D structure of the target protein is unknown, computational methods (such as comparative modeling (Lemer et al., 1995), fold recognition (Jones et al., 1992), or *ab initio* or *de novo* modelling (Lesk and Chothia, 1980; Simons et al., 1997)) can be used to predict the structure from its sequence. Next, the energy minimization step optimizes unreasonable conformations in the initial structure to avoid system collapse during the simulation. Subsequently, the equilibrium simulation gradually adjusts temperature and pressure to bring the system to a state of thermodynamic equilibrium. Finally, in the production simulation phase, data is collected under equilibrium conditions for the analysis of dynamic behaviors (such as conformational changes, binding free energy, etc.) (Figure 2; Lei and Duan, 2008; Ulmschneider and Ulmschneider, 2018; Chen et al., 2020). To assess the stability and interactions of protein-ligand complexes, several indicators are commonly used. Root Mean Square Deviation (RMSD) measures the extent of structural deviation in the protein-ligand complex; lower RMSD values indicate greater structural stability. Root mean square fluctuation (RMSF) measures the flexibility of residues, where high fluctuations may destabilize the binding site. Radius of Gyration (Rg) assesses the compactness of the complex. MM/PBSA or MM/Generalized Born Surface Area (GBSA) calculates the binding free energy, providing quantitative estimates of binding affinity. For instance, the binding stability of the LRGFGNPPT peptide with the targets AKT serine/threonine kinase 1 (AKT1), angiotensin-converting enzyme (ACE), and renin (REN) was confirmed by 200 ns MD simulation (Gao et al., 2023; Chen et al., 2024; Hadi et al., 2025). Currently, common MD simulation software (such as AMBER, CHARMM, GROMACS, NAMD and LAMMPS) have versions released that support GPUs computing (Table 2), significantly enhancing simulation efficiency and providing strong support for the dynamic studies of complex systems. Model-driven MD relies on human-established physical formulas for calculating forces. In contrast, data-driven MD uses large datasets and machine learning to model potential energy. The former is interpretable, but it is challenging to extend beyond its predefined functional form. The latter offers high accuracy but requires a large amount of data and validation of its extrapolation capabilities (Jo et al., 2017; Shirts et al., 2017; Fiorin et al., 2024; Kedir et al., 2024).

**TABLE 2:** Commonly used MD simulation tools.

Tools	Description	Availability	Link	Ref.
AMBER	A force field and simulation tool focused on biological macromolecules	Partially free	http://ambermd.org/	Case et al. (2005)
CHARMM	A software tool that supports multiple force fields and simulation conditions	Commercial	http://www.charmm.org/	Brooks et al. (2009)
GROMACS	An open-source software with efficient parallel computing capabilities	Free	https://www.gromacs.org/	Makarewicz and Kaźmierkiewicz (2013)
NAMD	A parallel software suitable for large-scale simulation of biological molecular systems	Free	https://www.ks.uiuc.edu/Research/namd/	Phillips et al. (2005)
LAMMPS	A large-scale parallel software applicable to materials science	Free	https://lammps.sandia.gov/	Panwar et al. (2023)

MD simulation plays a key role in drug development. Firstly, by looking at simulation data, this method can evaluate how stable the predicted binding modes are and give detailed dynamic information at the atomic level. Researchers can see if key interactions stick around in a changing environment and can also discover hydrogen bonds that involve water or new interaction sites that were not considered in the initial conformation. These findings are crucial for making targeted changes to molecular structures, which helps improve how drugs interact with their targets. Secondly, MD simulation can precisely calculate binding free energies, helping to pinpoint which small structural tweaks can really boost affinity. This lets researchers focus on the most promising molecules for synthesis and experimental testing, which really speeds up drug optimization and helps reduce the emergence of drug resistance (Hu et al., 2011; Zhang et al., 2020). However, this method does have its downsides. Running simulations that last microseconds or longer needs powerful computing clusters and substantial time, which really limits how much can be done. Therefore, MD simulation is usually only good for digging deep into a few top candidate molecules rather than large-scale screening. Moreover, the reliability of the simulation results is highly dependent on the accuracy of the chosen force field parameters. Existing force fields might not accurately describe non-standard residues, metal ions, or special chemical bonds, which can mess with the accuracy of the final predictions (Merelli et al., 2007; Klepeis et al., 2009).

## 3 Application of the four core technologies in cancer drug development

### 3.1 Application of omics strategies in cancer drug development

Omics strategies hold significant application value in cancer drug development. Furthermore, by integrating multiple omics technologies (such as genomics, proteomics, metabolomics, etc.), researchers can deeply analyze the molecular mechanisms of cancer from multiple levels, thereby accelerating the discovery of drug targets, drug screening, and the formulation of personalized treatment plans (Dhuli et al., 2023; Nema, 2024; Yan et al., 2025).

Omics strategies provide efficient research pathways for drug target discovery. By analyzing 14 cancer subtypes in TCGA multi-omics dataset, a study revealed 40 driver genes associated with the Wnt, Notch, Hedgehog, JAK/STAT, NK-KB, and MAPK signaling pathways. In addition, by integrating WES, WGS, RNA sequencing (RNA-seq), miRNA, and DNA methylation analysis, along with high-resolution mass spectrometry proteomics data, researchers successfully classified Lung Adenocarcinoma (LUAD) into four subtypes with different clinical and molecular characteristics, discovering unique mutation features associated with specific populations (Heo et al., 2021). In the field of colorectal cancer treatment, Surachai Maijaroen’s team discovered through innovative research that: 1 RT2 treatment significantly inhibits Caco-2 cell proliferation and induces apoptosis; 2 proteomics analysis based on label-free LC-MS/MS identified 1,044 differentially expressed proteins, including 133 downregulated proteins and 79 upregulated proteins. Further analysis showed that downregulated proteins were mainly enriched in cancer cell proliferation pathways, while upregulated proteins significantly participated in apoptosis regulation. By prediction through the Search Tool for Interactions of Chemicals (STITCH) database and validation with real-time quantitative PCR (qPCR), the study successfully identified 39 downregulated proteins with potential drug interactions. This research not only confirmed the therapeutic potential of RT2 as a novel anticancer agent but also revealed its molecular mechanism of inhibiting tumor growth through the precise regulation of protein expression networks (Maijaroen et al., 2022). Moreover, Wilson’s research team systematically analyzed the regulatory network of histone lysine demethylation by integrating transcriptomics and epigenetics data. They found that epigenetic regulatory factors (such as KDM1A, KDM3A, EZH2, and DOT1L) play key regulatory roles in promoting mitosis. These factors are not only involved in tumor occurrence and development but are also closely related to the formation of drug resistance, thus holding significant value for therapeutic target development (Filipp, 2017; Wilson and Filipp, 2018).

Omics strategies play an increasingly important role in drug screening. Research further integrates organoid drug screening with multi-omics technologies, systematically analyzing the mechanisms of drug action via transcriptome sequencing and proteomics analysis of drug-treated organoid samples (Toshimitsu et al., 2022; Shin, 2023; Singh et al., 2025). For example, for the screened candidate drugs, researchers identified key genes and protein networks related to drug response through differential expression analysis, and combined phosphoproteomics to reveal the dynamic regulation of downstream signaling pathways (Toshimitsu et al., 2022). Furthermore, single-cell RNA-sequencing (scRNA-seq) technology (Delaney, 2021) was used to analyze the cellular heterogeneity of organoids under drug action, discovering specific molecular markers of resistant subpopulation, thereby guiding the optimization of combination therapy strategies (Chen et al., 2018; Nojima et al., 2025). This multi-omics-driven organoid platform not only validates the effectiveness of drug targets but also predicts drug-metabolite interaction networks through metabolomics analysis, providing a theoretical basis for dose design in subsequent clinical trials (Chen et al., 2018; Bigot et al., 2024). The research team also utilized epigenomic data (such as assay for transposase-accessible chromatin by sequencing (ATAC-seq)) to explore drug-induced changes in chromatin accessibility (Zou et al., 2021), discovering that epigenetic regulatory factors (such as Histone Deacetylases) may become synergistic targets to enhance drug sensitivity (Ramaiah et al., 2021). Ultimately, this strategy completes the full chain of precision research from drug screening to mechanism validation by integrating organoid functional experiments with multi-omics molecular maps (Mao et al., 2024).

In terms of the advancement of personalized medicine, the rapid development of omics technologies has had a profound effect. In the field of genomics, by analyzing key information such as individual-specific target variations, mutation loads, complex mutation characteristics, and tumor-specific antigens, a solid theoretical foundation has been laid for the three pillars of precision medicine—targeted therapy, immune checkpoint inhibitors, and personalized anticancer vaccines (Miao and Van Allen, 2016; Shibata et al., 2021; Song and Zhang, 2022). This technological breakthrough has rendered the molecular defect characteristics of tumor cells the groundwork for clinical diagnosis and treatment, significantly enhancing the scientific and precise nature of treatment plans. For example, the successful approval of trastuzumab for Human Epidermal growth factor Receptor 2 (HER2)-amplified metastatic BC and imatinib for BCR-ABL fusion-positive chronic myeloid leukemia marks the formal entry of cancer treatment into a new era of precision medicine (Druker et al., 2001; Slamon et al., 2001). Meanwhile, liquid biopsy technologies based on blood or plasma, especially by means of analyzing circulating free DNA (cfDNA) and circulating tumor DNA (ctDNA), have shown significant value in the diagnosis of BC, disease-free survival (DFS) (Silva et al., 2002) prediction, and the assessment and personalized management of triple-negative breast cancer (TNBC) (Ortolan et al., 2021; Neagu et al., 2023). With the iterative upgrade of high-throughput sequencing technologies, the deep integration of RNA-seq technology with bioinformatics methods and standardized databases has opened up new avenues for developing novel cancer biomarkers. Among them, research on alternative polyadenylation (APA) as a potential biomarker has made breakthrough progress. The integration of APA biomarkers with gene expression profiles, clinical covariates, and other traditional prognostic indicators is expected to construct a more precise diagnostic system, thereby promoting a qualitative leap in personalized cancer treatment (Kandhari et al., 2021). These technological advancements not only deepen our understanding of the molecular mechanisms of tumors but also provide more efficient and precise tools for clinical practice, marking the arrival of a comprehensive intelligent medical era (Fahmi et al., 2022; Long et al., 2023).

### 3.2 Application of bioinformatics in cancer drug development

Bioinformatics as an interdisciplinary field plays a key role in drug development. Its main contributions are reflected in three aspects: first, by systematically integrating multidimensional biological data, it can accurately identify potential drug targets and prognostic biomarkers (Jiang and Zhou, 2005; Cisek et al., 2016); second, by leveraging powerful data analysis capabilities, it can effectively elucidate and validate drug mechanisms of action (Gong et al., 2018); finally, it holds significant application value in predicting drug resistance (Rosati and Giordano, 2022).

Bioinformatics can systematically integrate multidimensional biological data, and given its powerful data analysis capabilities, accurately identify potential drug targets and prognostic biomarkers. Wang et al. conducted bioinformatics analysis using Oncomine, Bc-GenExMiner v4.3, PrognoScan, and UCSC Xena, discovering that the expression of the PITX1 gene is upregulated in BC and its low expression is associated with good patient prognosis, suggesting it may be a prognostic biomarker for BC. However, the reasons and mechanisms for its upregulation in BC require further study (Wang et al., 2020). Hossein Hozhabri et al. used TCGA, The University of Alabama at Birmingham Cancer (UALCAN), Kaplan–Meier plotter, The Breast cancer Gene-Expression Miner v4.7 (bc-GenExMiner v4.7), cBioPortal, STRING, Enrichr, MethSurv, and Tumor IMmune Estimation Resource (TIMER) for bioinformatics analysis, to find that high levels of CCL 4/5/14/19/21/22 were associated with better overall survival (OS) and recurrence-free survival (RFS), while elevated expression of CCL 24 was associated with shorter OS in BC patients. Additionally, high levels of CXCL 9/13 indicated longer OS, while increased expression of CXCL 12/14 was associated with better OS and RFS in BC patients. Conversely, increased transcription levels of CXCL 8 were associated with poorer OS and RFS in BC patients. The study results further indicated that CCL 5, CCL 8, CCL 14, CCL 20, CCL 27, CXCL 4, and CXCL 14 were significantly associated with clinical outcomes in BC patients. Thus, these findings provide a new perspective that may assist in the clinical application of CC and CXC chemokines as prognostic biomarkers for BC (Hozhabri et al., 2022). Hu et al. utilized various bioinformatics tools to analyze the expression of Zinc Finger Protein 207 (ZNF 207) in various cancer samples from the TCGA database, such as TIMER 2.0, cProSite, UALCAN, the SangerBox analysis tool, the Gene Expression Profile Interactive Analysis 2 (GEPIA2) tool, the Tumor-Immune System Interaction Database (TISIDB) portal, and the Tumor Immune Dysfunction and Exclusion (TIDE) platform. The upregulated expression of ZNF 207 was found to be associated with poorer prognosis and play an important role in immune evasion and the tumor microenvironment. Notably, ZNF207 has been identified as a potential prognostic biomarker and therapeutic target in liver cancer; however, future studies need to further elucidate the specific molecular mechanisms of ZNF207 to fully utilize its potential in cancer treatment (Hu et al., 2024). By mining gene expression profile data, mutation data and other relevant information, differential genes and proteins closely related to cancer occurrence and development can be screened, which may serve as potential drug targets and prognostic biomarkers, providing key clues for the precise treatment of cancer (Xu et al., 2021; Lin et al., 2022).

In elucidating and validating the mechanisms of action of drugs, bioinformatics utilizes technologies such as NP, molecular docking and MD simulation to simulate interactions between drugs and targets, constructing drug-target networks, predicting drug action targets and pathways, and further analyzing mechanisms of action, providing theoretical support for drug development and clinical applications (Dahl et al., 2002; Rajadnya et al., 2024). At the same time, using bioinformatics tools to dynamically monitor and analyze gene expression changes and signaling pathway regulations during drug action helps to further validate drug mechanisms of action and optimize drug treatment plans (Alshabi et al., 2019). Gong et al. first utilized bioinformatics tools such as DrugBank 5.0 to screen 11 direct protein targets (DPTs) of aspirin and deeply analyzed the protein interaction networks and signaling pathways of these DPTs, discovering that aspirin was associated with various cancers, with small cell lung cancer (SCLC) being the most closely related. Subsequently, through the cBio Portal, detailed classifications of mutations in four aspirin DPTs (IKBKB, NFKBIA, PTGS2, and TP53) in SCLC were performed. Meanwhile, ONCOMINE was used to identify the top 50 overexpressed genes in SCLC, and STRING was further utilized to identify genes related to the four aspirin DPTs in SCLC, ultimately discovering five consistent genes that may serve as potential therapeutic targets for aspirin in SCLC. This study revealed that aspirin exerts antitumor effects by intervening in the cell cycle regulatory network and activating the P53 signaling pathway, with mechanisms involving the regulation of cyclin-dependent serine/threonine kinase activity, thereby interfering with the proliferation process of SCLC cells. This finding not only provides a new perspective for elucidating the molecular mechanisms of aspirin in SCLC but also lays a theoretical foundation for developing combination therapy schemes based on cell cycle targeting (Gong et al., 2018).

In terms of predicting drug resistance, bioinformatics also demonstrates enormous application potential. By analyzing tumor cell gene mutations, gene expression profiles, and polymorphisms of drug metabolism-related genes, resistance prediction models can be established to predict tumor resistance to drugs, providing important evidence for clinicians to formulate personalized treatment plans, thereby improving the precision and effectiveness of cancer treatment and enhancing patient prognosis (Banerji et al., 2015; Olivier et al., 2019). For example, Diletta Rosat et al. utilized scRNA-seq and bioinformatics analysis to explore cancer heterogeneity and drug resistance mechanisms, employing specific clustering algorithms (such as k-means) to group cells and categorizing cells with similar gene expression patterns into the same class, thereby identifying different cell subpopulations. Tumor cells were classified into proliferative subpopulations, quiescent subpopulations and stem-like subpopulations on gene the basis of the expression characteristics, with different subpopulations potentially related to cancer resistance. Using differential expression analysis, specific genes expressed between different cell subpopulations can be further identified, which may play key roles in cancer resistance mechanisms. Furthermore, gene expression differences between pre-treatment and post-treatment or resistant and sensitive cell populations can be established to screen for differentially expressed genes (DEGs). These DEGs may be involved in the response of cancer cells to drugs, such as drug uptake, metabolism, target expression, and apoptosis regulation. Analyzing the functions and related pathways of DEGs helps to reveal the molecular mechanisms of cancer resistance (Rosati and Giordano, 2022). For example, in non-small cell lung cancer, the KRAS G12D mutation is associated with resistance, and through scRNA-seq and bioinformatics analysis, it was found that cell subpopulations with different KRAS expression levels respond differently to drugs. Patient-derived xenograft (PDX) cells that survived after treatment with various anticancer drugs showed lower risk scores (RS), lower KRAS expression levels, lower activation states of the RAS-MAPK signaling pathway, and significantly different expressions of ion channel transport genes, which may be related to drug resistance mechanisms (Kim et al., 2015).

### 3.3 Application of network pharmacology in cancer drug target development

NP, as an emerging research approach, has the advantage of considering multiple targets and signaling pathways simultaneously, which aligns with the complexity and heterogeneity of cancer. Therefore, by constructing drug-target-pathway networks, it provides a series of systematic strategies for cancer drug screening, focusing on its applications in target identification, drug repositioning and drug combinations (Azmi and Mohammad, 2014; Wang J. et al., 2022; Fatima et al., 2024).

In NP, the key steps of screening potential drugs involve the construction and analysis of drug-target networks. First, it is necessary to collect information on drugs and their targets from existing biological databases, including known drug targets and potential targets obtained through predictions. Then, network models are constructed using visualization tools like Cytoscape and network topology analysis is performed. By analyzing indicators such as node degree and betweenness centrality, key drug and target nodes can be identified, revealing the mechanisms of action and potential therapeutic targets of cancer drugs (Gogoi and Saikia, 2022; Xiong et al., 2022). Following this workflow, Liu et al. screened 11 genes related to the immune-inflamed phenotype (IIP) prognosis of colorectal cancer patients from the targets of Yi-Yi-Fu-Zi-Bai-Jiang-San (YYFZBJS) through NP analysis and bioinformatics analysis (PIK3CG, C5AR1, PRF1, CAV1, HPGDS, PTGS2, SERPINE1, IDO1, TGFB1, CXCR2, and MMP9). They found that YYFZBJS can activate the immune response in colorectal cancer and alleviate inflammation in the IIP microenvironment, providing new perspectives for finding new therapeutic targets for traditional Chinese medicine and accurate diagnostic indicators for targeting tumors (Liu Y. et al., 2023). Bai et al. performed comprehensive pharmacological strategies combining NP, liquid chromatography-quadrupole time-of-flight tandem mass spectrometry (LC-Q-TOF/MS), and experimental analysis to demonstrate that Jiawei Xiaoyao Wan (JXW) exerts dual therapeutic effects on BC combined with depression in mice via multiple targets (TP53, ESR1, VEGFA, AKT1, IL6, TNF, EGFR). The potential mechanisms were found to be related to regulating neurotransmitter (5-Hydroxytryptamine) and inflammatory factor levels, and importantly, blocking the JAK2/STAT3 signaling pathway effectively alleviated depressive symptoms in BC combined with depression mice, inhibiting tumor progression (Bai et al., 2024). Furthermore, the analysis of drug-target networks not only facilitated drug repositioning but also revealed interactions between different drugs, providing a basis for finding potential drug combinations (Kumar and Roy, 2023).

Researchers can identify new uses for drugs that are already available. They do this through in-depth analysis of drug targets and mechanisms of action through NP (Azmi, 2013; Song et al., 2019). In BC research, drug repositioning is considered a promising approach to enhance clinical efficacy (Malik et al., 2022). HTS, QSAR, and NP technologies are widely applied to discover drugs for new indications (Chen et al., 2017; Satish et al., 2024). Recent studies have shown that drugs such as metformin, itraconazole and pimobendan have been repositioned to regulate metabolic and epithelial-mesenchymal transition (EMT) pathways, effectively inhibiting BC progression (Kandasamy et al., 2024). These studies underscore that drug repositioning can not only expand the indications of existing drugs but may also enhance the efficacy of cancer treatment through multitarget approaches, reduce side effects, and promote the development of combination therapies (Turanli et al., 2018; Turanli et al., 2021).

Combination therapy is an important strategy for improving anticancer efficacy and overcoming drug resistance (Kumar et al., 2022; Tufail et al., 2022). NP can predict potential drug combinations by analyzing the synergistic effects of drug combinations (Liu et al., 2017). Using synergistic drug combination prediction algorithms, such as multi-objective evolutionary algorithms and random search algorithms, synergistic drug combinations can be rapidly screened from large-scale drug combination spaces (Zinner et al., 2009). In addition, by constructing drug combination networks, the efficacy and safety of different drug combinations can be analyzed, providing guidance for clinical trials. For instance, combinations of paclitaxel, docetaxel, trastuzumab, and pertuzumab can effectively treat HER2-positive metastatic BC (Kawajiri et al., 2015; Tian et al., 2017). Antonio Federico et al. proposed an integrated NP framework that systematically prioritizes drug combinations with synergistic effects while considering the molecular characteristics of diseases and the intrinsic properties of drugs. This method is not only applicable to cancer but can also be extended to other complex diseases, providing new computational tools for drug repositioning and combination therapy. The researchers found that combinations of paclitaxel with non-cancer drugs (such as chlorpromazine) showed potential therapeutic value across various cancer types, warranting further experimental validation. Paclitaxel is the most commonly used drug across cancer types, especially in BC, prostate cancer and lung squamous cell carcinoma, and is often combined with chlorpromazine and carboplatin. Additionally, the specific combination of temozolomide with Aurora kinase inhibitors provides new therapeutic strategies for liver cancer, while the specific combination of navitoclax with psychiatric drugs (such as pentobarbital) offers new hope for colon cancer patients (Federico et al., 2022).

### 3.4 Application of molecular dynamics simulation in cancer drug target development

Based on the earlier technical methods, MD simulation allows researchers to identify potential drug binding sites, analyze conformational changes of drug molecules, and assess the stability and activity of molecules under specific conditions, which is crucial for optimizing drug design (Dore et al., 2017). Furthermore, MD simulation can be combined with other computational methods, such as molecular docking and VS, to provide comprehensive support for drug development (Kokh et al., 2016; Platania and Bucolo, 2021; Sharma et al., 2021; Trezza et al., 2025).

One of the most critical aspects of the drug development process is the interaction between drugs and targets. Through MD simulation, researchers can gain in-depth understanding of how drug molecules bind to their targets (such as proteins) and affect their functions. In research targeting the c-Met receptor tyrosine kinase (MET), two plant compounds were identified through VS and MD simulation, that is, neogitogenin and samogenin, which have significant binding affinity and show promising inhibitory activity against MET (Elasbali et al., 2024). Shams Tabrez et al. obtained the 3D structure of glutaminase (GLS) from the protein database and minimized it, screening approximately 60,000 natural compounds from a traditional medicine database, and prepared ligands using the “Prepare Ligand Module” of Discovery Studio 2020 (Ehrman et al., 2007). A control compound, 6-Diazo-5-Oxo-L-Norleucine, was retrieved from the PubChem database. Utilizing VS and molecular docking techniques, candidate compounds with high binding energy to GLS were identified and their stability was verified through MD simulation. GROMACS 5.1.2 was used with 100 ns MD simulation for GLS-DON (control), GLS-ZINC32296657 and GLS-ZINC03978829, with average RMSD (Sticht et al., 1994) values of 0.25, 0.33 and 0.31 nm, respectively. RMSD is an indicator of protein stability, with lower deviations indicating more stable protein structures. The results showed that all three formed stable complexes with GLS and exhibited hydrogen bonding interactions, with GLS-DON and GLS-ZINC03978829 showing relatively lower average RMSD values, indicating better binding stability and a higher abundance of hydrogen bonds. RMSF and solvent-accessible surface area (SASA) (Broz et al., 2024) analyses indicated that DON and ZINC03978829 interacts more closely with GLS, reducing the fluctuation of enzyme residues and apparent exposure. GLS-DON and GLS-ZINC03978829 formed 2-5 hydrogen bonds with the GLS catalytic pocket, while GLS-ZINC32296657 formed 1-2 hydrogen bonds. The energy landscape revealed that the GLS-ZINC03978829 complex has two distinct global energy minima basins, indicating greater stability. ZINC03978829 and ZINC32296657 had higher binding energies to GLS than the control compound 6-Diazo-5-Oxo-L-Norleucine, and MD simulation suggested that they form stable complexes with GLS, potentially serving as GLS inhibitors for cancer treatment, but the need for further experimental optimization was prompted (Tabrez et al., 2022).

Drug optimization, as a core aspect of drug development, aims to enhance the three key attributes of drugs through molecular structural modifications: biological activity, target selectivity and clinical safety. MD simulation technology exhibits unique advantages in this field, primarily reflected in two aspects: 1 Optimization of target binding characteristics. Through high-precision MD simulation, researchers can dynamically analyze the binding modes of drug-target complexes, accurately identifying key amino acid residue networks that influence binding affinity (Shen et al., 2013; Tripathi et al., 2016). A typical case is the development of glycogen synthase kinase-3β (GSK-3β)/inhibitor of nuclear factor-κB kinase-β (IKK-β) dual-target inhibitors, where the research team combined molecular docking with 200 ns MD simulation, not only verifying the binding stability of lead compounds but also discovering their unique hydrogen bond networks formed with the adenosine 5′-triphosphate (ATP) binding pockets of both targets, ultimately achieving half maximal inhibitory concentration (IC_50_) values of 12 nm and 8 nm for GSK-3β and IKK-β, respectively (Roy Acharyya et al., 2022). 2 ADME property prediction MD simulation can construct dynamic behavior models of drug molecules in physiological environments, quantitatively predicting absorption, distribution, metabolism, and excretion characteristics by calculating parameters such as ligand-membrane protein interactions and binding free energies of metabolic enzymes (Zheng et al., 2021; Çakır et al., 2025). This physics-based prediction method offers better interpretability than traditional QSAR models. This integrated MD simulation optimization strategy reveals structure-activity relationships at the atomic scale, significantly improving the success rate of drug design, which has therefore become an important paradigm in innovative drug development (Wade and Salo-Ahen, 2019).

## 4 Conclusion and outlook

The development and application of cancer treatment drugs are undergoing a revolutionary change. With the cross-integration of fields such as omics technologies, bioinformatics, NP, and MD simulation, personalized cancer treatment is showing incredible potential. These technological breakthroughs not only broaden research perspectives, but also provide innovative ideas for clinical treatment. However, the field still faces significant challenges, such as data heterogeneity, poor algorithm interpretability, and issues with clinical translation efficiency. Integrating cutting-edge technologies like AI, machine learning (ML), and deep learning (DL) is expected to overcome the limitations of traditional therapies, achieving more precise and efficient treatment plans.

At the application level, omics technologies generate massive amounts of data, but data heterogeneity restricts target identification efficiency. AI technologies can quickly analyze multi-omics data, can automatically extract features, and can predict drug-target interactions. Additionally, bioinformatics relies on computational tools to analyze biological data, while ML can optimize the generalization ability of ML algorithms, reducing prediction bias caused by overly simplified algorithms (Sarvepalli and Vadarevu, 2025). For example, DENVIS, a scalable algorithm that uses GNN, has demonstrated speed and accuracy advantages in compound screening (Krasoulis et al., 2022). NP focuses on multi-target drug interactions; however, it relies on experimental validation to avoid false-positive results. By applying DL technology, recent research has successfully identified potent discoidin domain receptor tyrosin kinase 1 (DDR1)inhibitors, illustrating how AI can accelerate the identification and validation of specific drug targets in cancer treatment (Zhavoronkov et al., 2019; Talevi et al., 2025). AI predicts drug-target-disease associations by analyzing large-scale biological networks (such as PPI networks), aiding drug repurposing and significantly reducing research and development costs (Tran et al., 2023; Wang et al., 2023). In drug screening, the combination of VS and AI can accelerate drug discovery, with the AlphaFold model enabling the prediction of protein structures that facilitated the identification of a new candidate drug (ISM042-2-048) targeting cyclin-dependent kinase 20 in liver cancer cells in the absence of experimentally determined target protein structures (Ren et al., 2023; Wang et al., 2024). Molecular docking technology combined with AI can identify compounds that are more effective against specific targets (Batool et al., 2019; Vamathevan et al., 2019). Platforms like RosettaVS have been developed to predict docking poses and binding affinities by using enhanced force fields and flexible receptor models to improve prediction accuracy. This method can simulate conformational changes occurring upon ligand binding, which is crucial for accurately predicting how different compounds interact with their targets (Zhou G. et al., 2024). MD simulation provides atomic-level binding details but are computationally expensive and parameter-sensitive. AI can significantly accelerate this process and improve accuracy. Integrated machine learning force fields (such as DeePMD) can reduce simulation time while maintaining accuracy. Additionally, DL models (such as three-dimensional convolutional neural network (3D-CNN)) can analyze trajectory data to predict dynamic changes at binding sites, effectively lowering computational costs and improving simulation accuracy (Peivaste et al., 2024; Zeng et al., 2025).

Despite the significant improvements in target discovery and drug design efficiency through the integration of multi-omics data, bioinformatics analysis, NP, and MD simulation in cancer drug development, the application still faces multiple limitations and challenges. First, model performance really depends on good, standardized multi-omics data; however, in reality, data often suffer from incompleteness, heterogeneity, and privacy protection restrictions; hindering cross-institutional data sharing and model generalization capabilities. Second, AI models (especially DL) generally face the “black box” problem, lacking interpretability and making it difficult to meet clinical and regulatory requirements for transparency of mechanisms and causal inference. Furthermore, bias in algorithm training may exacerbate health inequalities and hinder discovery of novel targets. From an ethical perspective, patient privacy, data de-identification, informed consent, and algorithmic bias have become key issues. Particularly when dealing with sensitive information such as genomics and transcriptomics, strict data governance and access mechanisms need to be established within the frameworks of regulations like the Health Insurance Portability and Accountability Act (HIPAA) and the General Data Protection Regulation (GDPR). In the future, we need to create an open, trustworthy, and ethically sound data-sharing system and to promote the development of explainable AI and physics-guided models to achieve the true implementation of AI-driven precision medicine in cancer.
